# Host origin is a determinant of coevolution between gene segments of avian H9 influenza viruses

**DOI:** 10.1128/jvi.01518-24

**Published:** 2025-06-13

**Authors:** Jennifer E. Jones, Seema S. Lakdawala

**Affiliations:** 1 Department of Microbiology & Molecular Genetics, University of Pittsburgh 6614, Pittsburgh, Pennsylvania, USA; 2 Center for Evolutionary Biology and Medicine, University of Pittsburgh 607640, Pittsburgh, Pennsylvania, USA; 3 Department of Pediatrics, University of Pittsburgh School of Medicine 12317 , Pittsburgh, Pennsylvania, USA; 4 Department of Microbiology & Immunology, Emory University 197280, Atlanta, Georgia, USA; Cornell University Baker Institute for Animal Health, Ithaca, New York, USA

**Keywords:** avian viruses, influenza, H9, reassortment, virus evolution, phylogenetics

## Abstract

Several emerging influenza viruses, including H7N9 and H5N6 viruses, trace their origins to reassortment with H9N2 viruses that contributed internal gene segments. However, the evolutionary constraints governing the reassortment of H9N2 viruses remain unknown. In seasonal human influenza A viruses, gene segments coevolve at both the nucleotide and amino acid levels. Here, we demonstrate that evolutionary relationships between gene segments, including polymerase subunits in human H3N2 viruses, differ from avian H9 viruses. Avian H9 viruses were characterized by little coevolution between gene segments or between polymerase subunits. Strikingly, protein trees built from avian H9 polymerase subunits diverge despite known functional constraints on polymerase evolution. The evolutionary divergence observed between gene segments of avian H9 viruses was consistent across isolates from different continents, suggesting that coevolution between H9 gene segments is not dependent on regionally defined avian lineages. Instead, coevolution between gene segments was only found in H9 viruses that crossed the species barrier into humans. Our study reveals the role of the host in the coevolution of influenza gene segments and suggests that high reassortment potential in avian species may be a consequence of evolutionary flexibility between gene segments.

**IMPORTANCE:**

Emerging pandemic influenza viruses can contain a combination of viral gene segments from avian, swine, and/or human species through the process of reassortment. H9 viruses have been important gene segment donors to several avian viruses of concern, including H5N1 and H7N9. In this work, we found that H9 gene segments and proteins do not have constrained evolutionary relationships typical of human seasonal influenza viruses, suggesting a flexibility that could allow for greater reassortment potential. However, we also found that this observation was dependent upon the host species source, with greater evolutionary constraints in H9 viruses from human sources. Understanding such constraints that underlie viral reassortment is critical to predicting future viruses that may be feasible in nature and have pandemic potential.

## INTRODUCTION

Host range restrictions influence viral emergence into the human population (1, 2). Influenza A viruses are found in a wide range of hosts, but the natural reservoirs are wild aquatic waterfowl and shorebirds (3, 4). Spillover of avian influenza viruses into humans is rare, but serious zoonotic outbreaks can occur when host range restrictions are overcome (4). In just one example, the H7N9 outbreak in mainland China, a triple reassortant virus with Eurasian H7 hemagglutinin (HA), wild bird N9 neuraminidase (NA), and six internal H9N2 gene segments, caused five successive epidemics between 2013 and 2017 with over 1,500 human infections and a mortality rate of 39% (5, 6). It is therefore of paramount importance to understand the evolutionary mechanisms that promote the emergence of avian influenza viruses in mammalian hosts.

Ecological challenges imposed by the host profoundly impact the spatiotemporal dynamics of viral evolution and emergence (1). Within migratory birds, avian influenza lineages are restricted by host species as well as the migratory routes frequented by these hosts, with little interhost reassortment detected (3, 7). In contrast, in domestic landfowl such as chickens and turkeys, influenza virus subtypes frequently cocirculate and efficiently reassort (8, 9). Adaptation and reassortment of H9N2 viruses in vaccinated farm chickens led to the emergence of the G57 genotype in 2007 with increased antigenicity that overtook all other genotypes by 2013 (10). H9N2 viruses in turn contributed gene segments to other cocirculating influenza viruses, including recently emerging H7N9, H5N6, and H10N8 viruses (5, 10–13). As of 2016, H9N2 viruses became the dominant subtype in both chickens and ducks in China (14). Therefore, it is critical to consider such factors as geographical restrictions imposed by migratory routes and host species in guiding the evolutionary mechanisms of avian influenza viruses.

Factors intrinsic to viruses also play a central role in viral evolution and emergence (1). Host range of an influenza virus is impacted by several viral properties, including receptor binding specificity and glycosylation of HA, stalk length of NA, and compatibility of the viral ribonucleoprotein (vRNP) complex with host factors such as ANP32A or nuclear translocation machinery (15, 16). Interactions between the viral glycoproteins (HA and NA) or between polymerase subunits (PB2, PB1, and PA) functionally constrain influenza virus evolution and can contribute to viral emergence (17, 18). Similarly, viral RNA packaging signals have been suggested to constrain the evolution of the gene segments themselves, likely through their role in vRNP assembly and packaging of segments into progeny virions (19–21). Consistent with this prior work, we recently demonstrated that gene-specific RNA-RNA interactions may also contribute to coevolution between gene segments in seasonal human influenza A viruses (22). However, whether evolutionary constraints imposed by physical interactions between viral RNA segments or viral proteins contribute to the evolution and emergence of avian influenza viruses remains unknown.

Here, we performed comparative genomics to investigate how the evolution of gene segments differs between human and avian influenza virus strains. We use our established methods to estimate coevolution between genes and proteins through a proxy tree distance calculation (22). Unexpectedly, we found minimal indication of coevolution between gene segments of avian H9 viruses. Instead, we provide evidence that the evolutionary trajectories of each gene segment coevolve in a host-specific manner in H9 viruses. Our study highlights the role of host origin in shaping influenza virus evolution.

## RESULTS

### Coevolution is observed between gene segments of seasonal human H3N2 viruses but not avian H9 viruses

**Fig 1:**
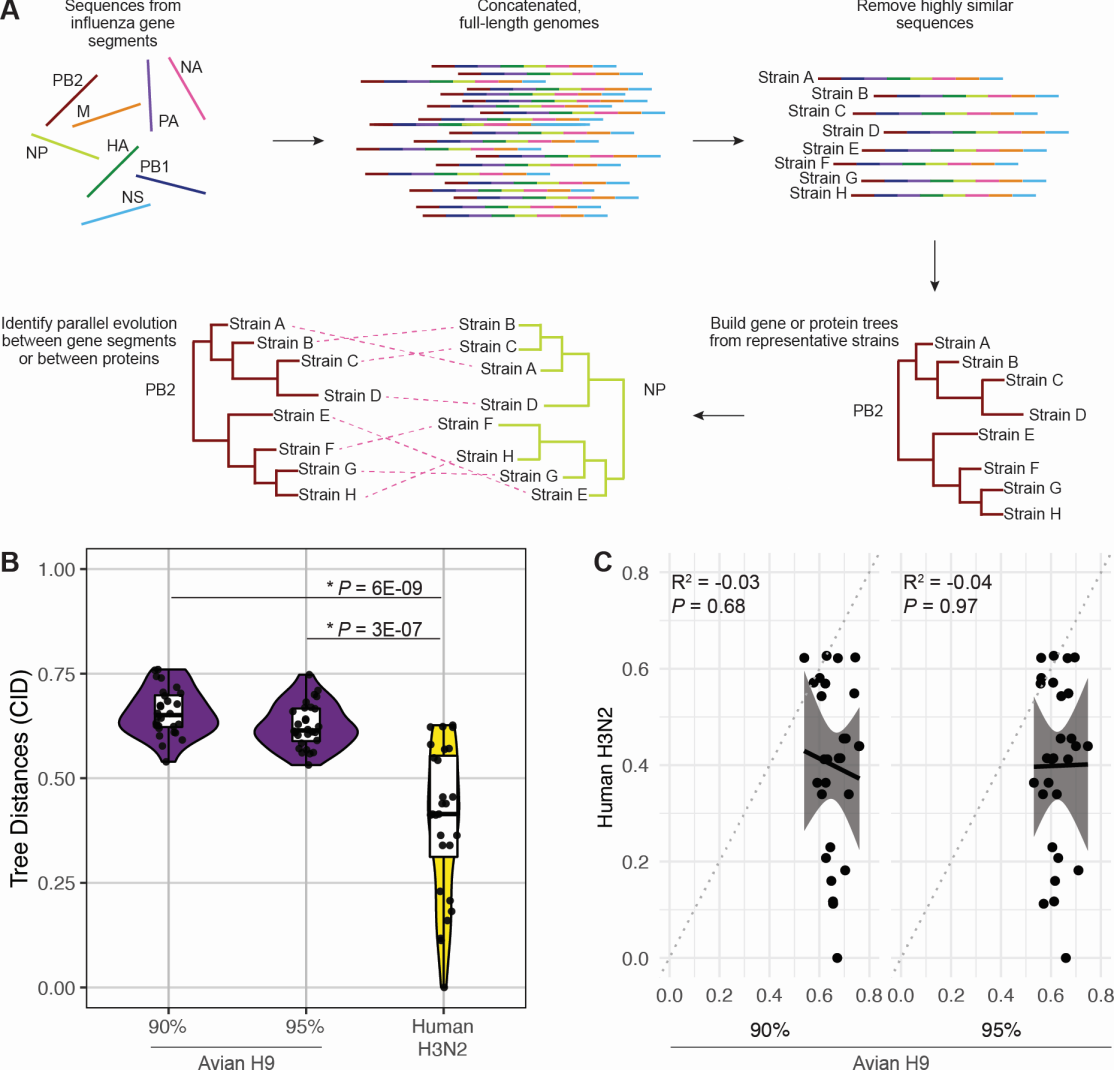
Minimal coevolution is found between avian H9 virus gene segments compared to human H3N2 viruses. (A) Sequences from avian H9 or human H3N2 virus gene segments were obtained from the Influenza Research Database. Gene segment sequences were concatenated into full-length genomes, and highly similar sequences were pruned. Maximum likelihood trees of each gene segment were reconstructed from representative strains. Protein trees were reconstructed from coding sequences. Tree similarity was assessed by quantifying the CID and used as a proxy for coevolution. (B) CID from avian H9 viruses (90% or 95% similarity thresholds as indicated; purple) compared to human H3N2 viruses (95% similarity threshold; yellow). Each point designates the distance between one pair of gene segment trees. Asterisks (*) indicate *P* < 0.05 (Mann-Whitney *U* test). (C) Linear regression was performed on pairwise CID. Solid line, best fit. Shaded region, 95% confidence interval. Dotted line, line of identity.

In our prior work, we studied whether gene segments of seasonal human influenza A viruses coevolve (22). Coevolution between gene trees was examined using publicly available human H1N1 and H3N2 virus sequences, as depicted in Fig. 1A. Tree distance, calculated by the clustering information distance (CID), was used to assess coevolution between genes, with the trees for any two gene segments considered coevolving if the tree distance between them was within the 95% similarity threshold. CID is measured from 0 to 1, with lower CID values indicating greater similarity between the gene segments and values approaching 1 indicating dissimilarity. Therefore, a tree with no constraints would return a value close to 1, and a gene tree compared to itself would return a value of 0. The extent of coevolution between pairs of gene segments from human seasonal influenza viruses varied widely, from robust coevolution with tree distances approaching 0 to large dissimilarity with tree distances of 0.6 (22). Coevolution between specific pairs of gene segments differed between H1N1 and H3N2 viruses but was relatively consistent over time in H3N2 viruses (22). Based on this observation, H3N2 viruses were selected as a benchmark for comparison to avian H9 viruses.

To explore whether coevolution between gene segments differs in H3N2 and H9 viruses, we took a similar approach to our previous study (Fig. 1A). Briefly, representative sequences are selected from genomic trees (i.e., species trees) by a sequence similarity threshold (see Materials and Methods for additional details). This approach significantly improves gene tree reconstruction by pruning highly similar sequences that do not resolve well in the tree (22). In this study, this approach offered the additional advantage of capturing similar degrees of diversity despite differing sequence availability in human and avian influenza sequences. When a 95% sequence similarity threshold was applied to both human H3N2 and avian H9 viruses, far more sequences remained in avian H9 virus alignments than in human H3N2 virus alignments (200 and 15 respective sequences; Table S1). These differences led to a disparity in tree size between human H3N2 and avian H9 viruses that could artificially inflate differences in tree distance. To account for this, two different sequence similarity thresholds were used to construct trees from avian H9 viruses (90% or 95%). When the sequence similarity threshold was reduced to 90% for avian H9 viruses, the number of unique clusters in the species tree fell from 200 to 56 (Table S1), making these trees more comparable in size to those constructed from human H3N2 viruses.

Following a modified version of our previously established workflow (22), gene trees were constructed for each of the eight gene segments of each set of viruses (Fig. S1 and S2 and data not shown). Coevolution between genes was determined by the CID value between each pair of gene trees. Similar to what we have previously reported (22), tree distances from seasonal human H3N2 viruses ranged from 0—indicating two identical trees—to 0.63 (Fig. 1B). In contrast, gene trees derived from avian H9 viruses exhibited significantly higher tree distances than gene trees derived from human H3N2 viruses, ranging from 0.53 to 0.76 (Fig. 1B). We found no correlation between pairwise CID from avian H9 viruses and human H3N2 viruses (*R*^2^ = 0.03–0.04, Fig. 1C). These differences were independent of the sequence similarity threshold applied during avian H9 virus gene tree reconstruction (Fig. 1B and C). Additionally, tree distances from avian H9 viruses lacked the variation seen in human H3N2 viruses, suggesting little to no preferential evolutionary relationships have formed between individual pairs of gene segments in avian H9 viruses. These data suggest that coevolution between gene segments is higher in human seasonal H3N2 viruses compared to avian H9 viruses.

### High evolutionary divergence is found between polymerase subunits of avian H9 viruses

**Fig 2:**
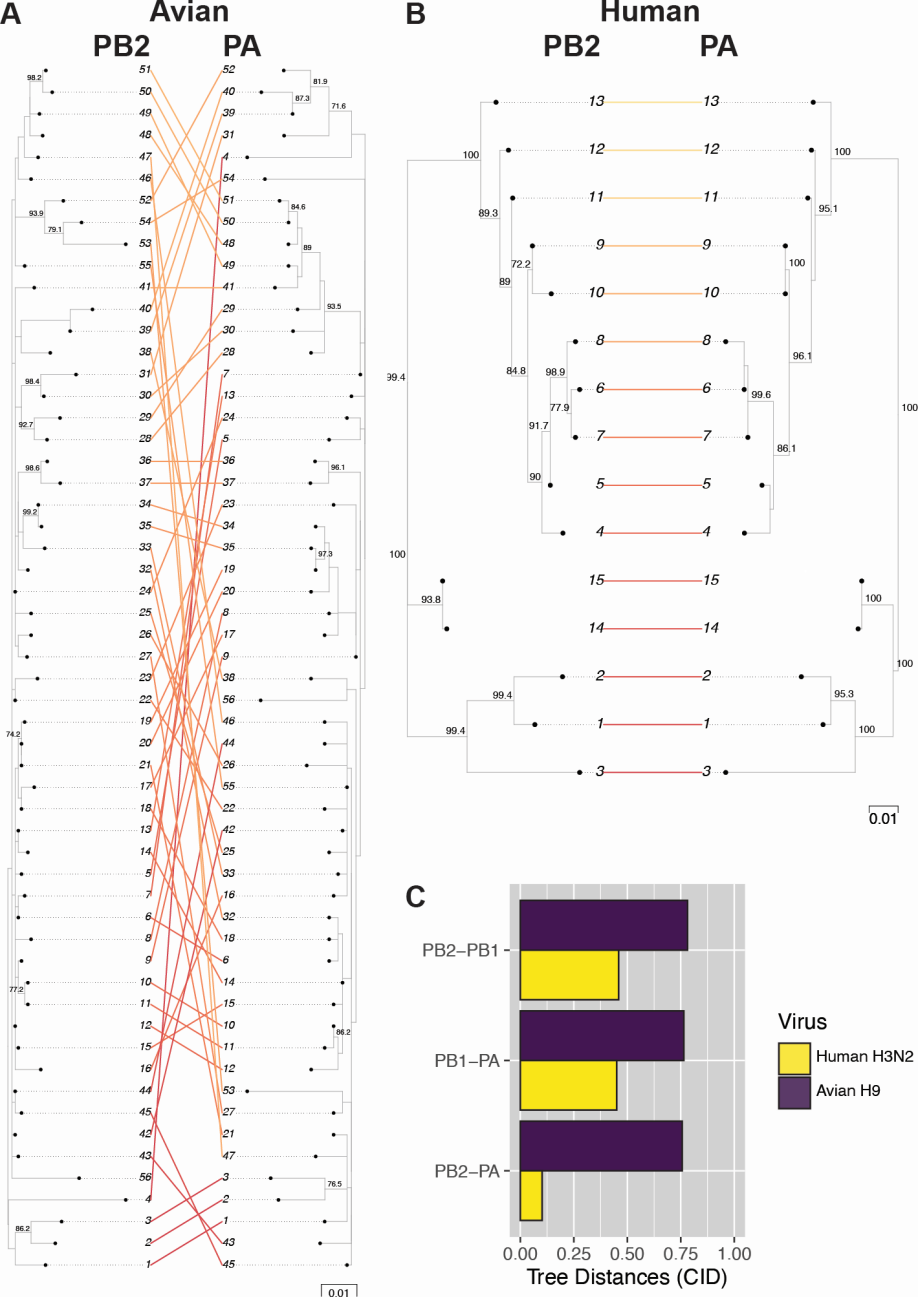
Avian H9 polymerase subunits do not exhibit coevolution. Coding sequences corresponding to the gene trees of avian H9 viruses and human H3N2 viruses were used to construct protein trees. (A and B) Tanglegrams visualizing tree similarity were constructed from the PB2 and PA protein trees for avian H9 (**A**) or human H3N2 (**B**) viruses. Strain names are coded by cluster number. (C) Pairwise CID for all combinations of polymerase protein trees.

Our initial examination of nucleotide sequences captures evolutionary constraints driven by interactions between protein complexes as well as RNA-RNA interactions. It is surprising that we observed uniformly high divergence between all gene segments in avian H9 viruses, particularly between genes encoding the viral polymerase subunits: PB2, PB1, and PA. The evolution of these three segments is thought to be functionally constrained by protein-protein interactions essential to polymerase function (17, 23, 24). To confirm that coevolution does not occur between polymerase subunits in avian H9 viruses, we examined the similarity between protein trees. Trees from amino acid sequences of PB2, PB1, and PA from either human H3N2 or avian H9 viruses were constructed (Fig. 2). As expected, protein trees corresponding to human influenza polymerase subunits were characterized by high coevolution, with tree distances ranging from 0.10 to 0.46 (Fig. 2B and C). In contrast, avian influenza polymerase tree distances were quite high, ranging from 0.76 to 0.78 (Fig. 2A and C). Thus, the polymerase subunits of avian H9 viruses do not exhibit coevolution, suggesting that greater flexibility in this protein complex may be tolerated in avian hosts.

### Divergence of H9 gene segments is consistent across geographical regions

Our investigation takes advantage of the breadth of avian influenza virus sequences available in public databases, but such broad analyses are not without disadvantages. Surveillance of avian H9 viruses is much lower than that of human H3N2 viruses (Table S1). Therefore, sampling bias could distort phylogenetic interpretation. Sampling of avian H9 viruses over time in our data set was inconsistent, with an inordinately high proportion of sequences coming from 2013 (Fig. S3A). The disproportionately high representation of sequences from this year coincides with the emergence of H7N9 viruses in China, reflecting heightened poultry surveillance efforts (5, 11). However, our strategy to select sequences from clustering mitigates this sampling bias, with 2013 isolates dropping from 25% of the overall data set to 13% of sequences selected for phylogenetic reconstruction (Fig. S3B). Therefore, it is unlikely that our results were greatly impacted by inconsistent surveillance over time.

**Fig 3:**
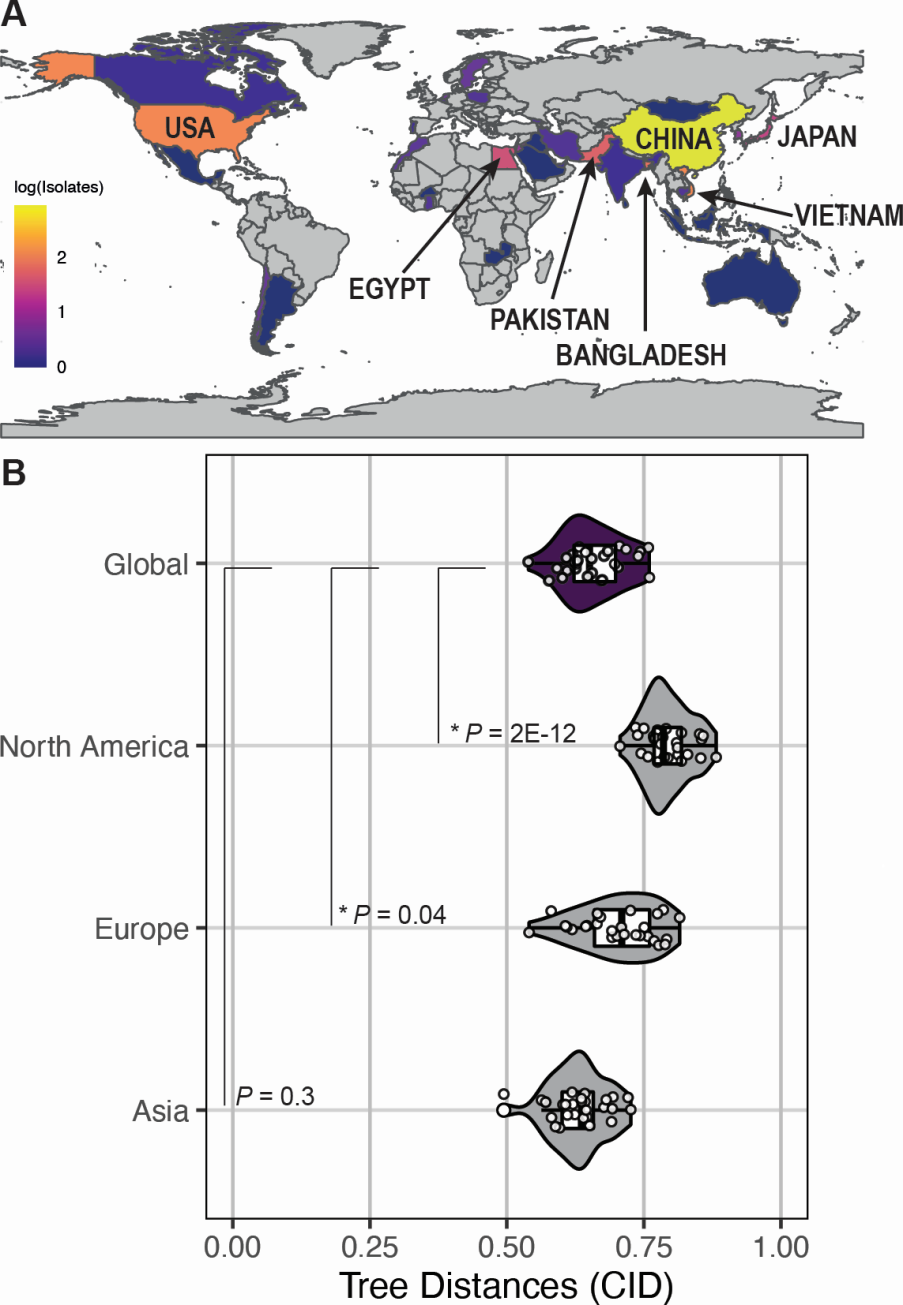
Avian H9 gene tree divergence is independent of region. (A) Global prevalence of avian H9 viruses. The total number of full-length sequences was log-transformed for visualization. Highest prevalence: China, 861; Vietnam, 120; USA, 118; Bangladesh, 72; Pakistan, 51; Egypt, 34; Japan, 25. (B) Avian H9 viruses were subset by continent, and representative sequences were chosen from each data set. Maximum likelihood trees of each gene segment were reconstructed from representative sequences. Continents with fewer than 10 clusters were excluded. Tree similarity was assessed as described in Fig. 1A. Each point designates the distance between one pair of gene segment trees. Asterisks (*) indicate *P* < 0.05 (Mann-Whitney *U* test with Benjamini-Hochberg *post hoc* correction).

Another important consideration for avian H9 virus evolution is geographical region. One plausible explanation for the apparent lack of coevolution between gene trees in H9 viruses is that coevolution between gene segments is lineage-specific. We previously reported a similar observation in H1N1 viruses isolated before and after the 2009 pandemic (22). Two geographically distinct H9N2 lineages have emerged in birds from North America and Eurasia (8). Therefore, we examined whether coevolution between avian H9 virus gene segments is regionally defined. The vast majority of avian H9 viruses were isolated from Asia (85%), primarily China (Fig. 3A). Of the remainder, roughly 9% of sequences were isolated from North America, 3% from Africa, 2% from Europe, and less than 1% from South America. We subset avian H9 viruses by continent of origin, excluding viruses from continents with fewer than 10 clusters (see Materials and Methods for additional details and Fig. S4 to S6 for representative trees). When avian H9 viruses were subdivided in this manner, no regional patterns in coevolution were detected (Fig. 3B). However, tree distances were in fact significantly higher in some regions compared to the global data set. This observation suggests that while lineage-specific differences in tree distances exist, minimal evidence of coevolution between gene segments is found in avian H9 viruses from any geographical location.

### Coevolution between H9 virus gene segments is dependent upon host origin

**Fig 4:**
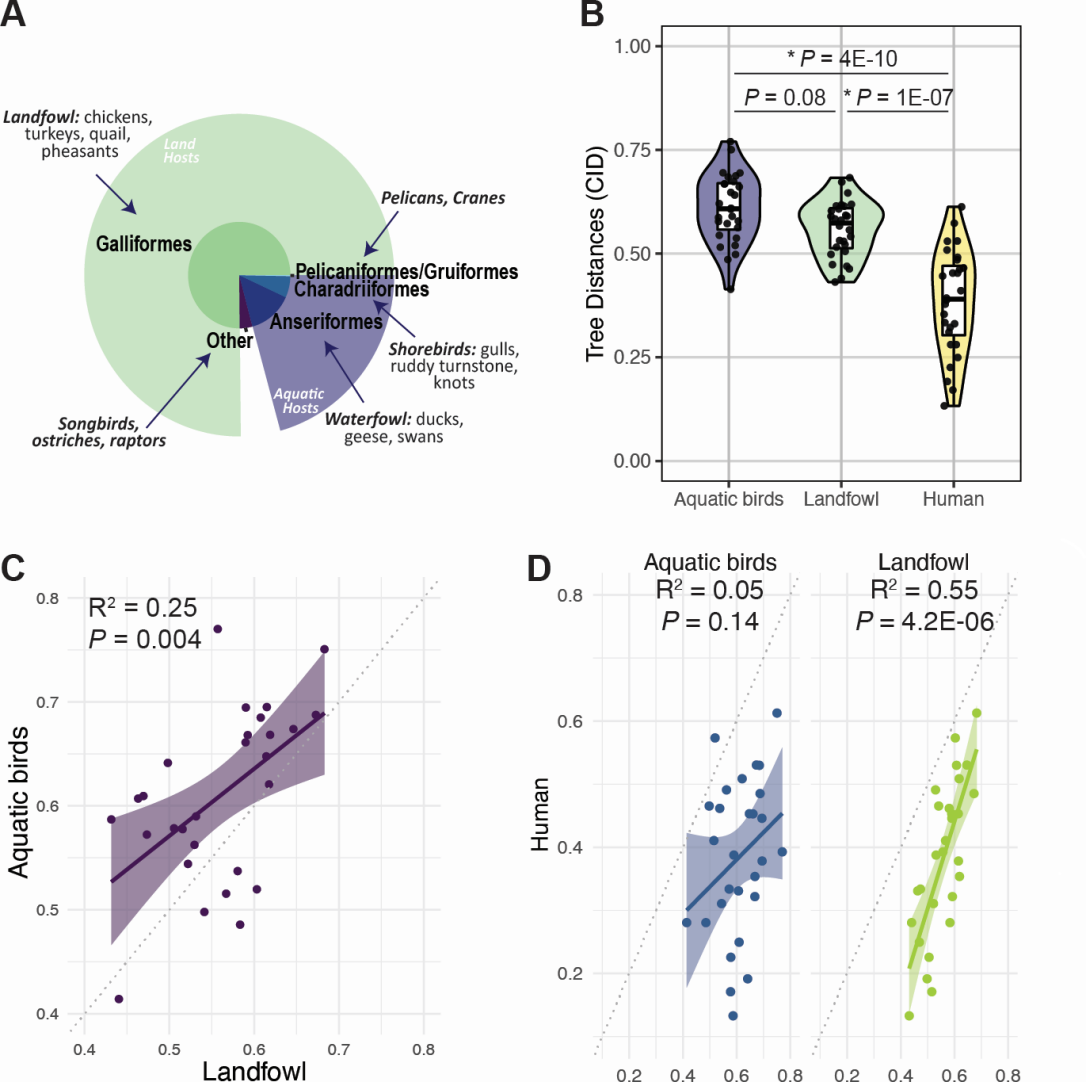
Coevolution between gene segments of H9 viruses is dependent upon host origin. (A) The distribution of H9 viruses across avian taxonomic orders. (B) CID values between all pair combinations of vRNA from Asian-origin H9 viruses isolated from aquatic birds, landfowl, or humans. Asterisks (*) indicate *P* < 0.05 (Mann-Whitney *U* test with Benjamini-Hochberg *post hoc* correction). (C and D) Linear regression was performed on pairwise CID. Solid line, best fit. Shaded region, 95% confidence interval. Dotted line, line of identity. (C) Comparison of CID in H9 viruses isolated from landfowl to CID in H9 viruses isolated from aquatic birds. (D) Comparison of CID in H9 viruses isolated from humans to H9 viruses isolated from aquatic birds or landfowl.

Given that geographical region did not contribute to coevolution between gene segments in avian H9 viruses, we examined whether host origin impacts coevolution. Host origin could play a role in the overall differences we observed in coevolution between gene segments in human H3N2 and avian H9 viruses (Fig. 1B and C). Additionally, previous studies suggest that reassortment in wild birds is restricted by host species (7). Therefore, we examined coevolution between gene segments of H9 viruses isolated from different hosts, including humans, landfowl, and aquatic birds. Aquatic birds are the natural reservoir of influenza viruses (4), but a sizeable majority of H9 viruses are also found in landfowl such as chickens, turkeys, and quail (Fig. 4A). Given that tree distances differed in H9 viruses isolated from different continents (Fig. 3B), we focused our analysis on H9 viruses isolated from Asia, where H9 sequences from humans, landfowl, and aquatic birds were all available. Tree distances from human H9 viruses were significantly lower than those obtained from H9 viruses from either set of avian hosts (Fig. 4B; Fig. S7 to S9). In addition, only tree distances from H9 viruses isolated from humans exhibited the wide range seen in human H3N2 viruses, suggesting coevolution between gene segments is host-dependent. We used linear regression to examine the degree of similarity between individual pairs of gene segments in H9 viruses from different hosts. To our surprise, tree distances from landfowl H9 viruses were more robustly correlated with those from human hosts than with those from aquatic birds (*R*^2^ = 0.55 vs 0.25) (Fig. 4C and D). In contrast, tree distances from H9 viruses from aquatic birds were not correlated with tree distances from H9 viruses from human hosts (*R*^2^ = 0.05) (Fig. 4D).

Tree distances between the PB2, PB1, and PA gene segments become smaller from aquatic birds to landfowl and again to human hosts (Fig. 4B, open circles). Most strikingly, the distance between the PB2 and PA trees is 0.695 in aquatic birds, 0.590 in landfowl, and 0.446 in human hosts. Similarly, the distance between the PB2 and PB1 trees falls from 0.695 in aquatic birds to 0.615 in landfowl and 0.378 in human hosts. The only pair of trees that did not follow this pattern was PB1 and PA, which remained consistent in aquatic birds and landfowl but dropped in human hosts (CID values of 0.414, 0.440, and 0.281, respectively). However, PB1 and PA shared the lowest distance among all three gene segments in all hosts. Altogether, these data suggest that coevolution between gene segments is dependent on host origin.

## DISCUSSION

The mechanistic underpinnings of viral evolution are complex. In the present study, we investigated the interplay between the host niche and functional constraints on influenza A virus evolution. Our results reveal that evolutionary constraints imposed by interactions between viral polymerase subunits in seasonal human influenza viruses do not extend broadly to avian H9 viruses. Instead, the coevolution of avian H9 polymerase subunit proteins is highly divergent. Surprisingly, the spatial structure of avian influenza lineages established by host migratory routes contributes minimally to the coevolution of avian influenza gene segments. Instead, gene segment coevolution in H9 viruses occurs in a host-dependent manner. These results highlight the impact of the host niche on influenza virus evolution.

Interactions between viral proteins have long been theorized to impose functional constraints on influenza virus evolution. Coordinated roles between the viral polymerase subunits are well-described and functionally constrain the evolution of each subunit in human influenza viruses (17, 22, 25). Surprisingly, we did not find evidence of coevolution between polymerase subunits in avian H9 influenza viruses in this study. However, coevolution between polymerase subunits was greater in H9 viruses isolated from humans than those found in avian species. This observation could be due to the adaptation of the polymerase segments in humans, or it could be due to sampling bias in the available sequences used in this analysis. A comparison of closely related avian strains to human isolates could reveal intermediate coevolutionary phenotypes in the polymerase subunit. Acquisition of substitutions in viral polymerase subunits that facilitate species jumps into the human population might require concomitant substitutions in other subunits to preserve function. Our results are consistent with prior studies demonstrating that the stability of the association between nucleoprotein and the viral polymerase in viral ribonucleoprotein complexes is critical for the evasion of innate immune sensing in human, but not avian, cells (26–29). Overall, these studies may suggest greater flexibility between viral polymerase subunits in avian hosts that allows for greater success of evolutionarily divergent viruses than in other species.

A lack of coevolution between influenza virus gene segments in avian H9 viruses may also have implications for genomic assembly. Selective packaging of all eight influenza gene segments is thought to occur through RNA-RNA interactions between gene segments (19–21, 30). We previously demonstrated that putative intersegmental RNA-RNA interactions could account for some coevolution observed between gene segments in human H3N2 viruses (22). Unlike in human H3N2 viruses, gene segments of avian H9 viruses are highly divergent. These results suggest that flexibility may exist in the genomic packaging of avian influenza viruses. Evolutionary plasticity in avian influenza gene segments could account for the increased reassortment frequency observed in influenza viruses in avian hosts (31). Further investigation may reveal a different mechanism of selective packaging of avian influenza gene segments altogether.

The problems posed by the unique ecological niche that domestic landfowl afford to avian influenza viruses are a growing concern. Influenza A viruses cocirculate in domestic landfowl and reassort efficiently in these hosts (8, 9). Moreover, the high incidence of coinfection of domestic landfowl with multiple influenza virus subtypes likely influences host range fitness trade-offs. Similar effects have been reported during coinfection with pepper mild mottle virus (1). Here, we discovered that coevolution between gene segments in H9 viruses isolated from landfowl more closely mirrors coevolution of gene segments from H9 viruses isolated from human hosts than aquatic birds. Our data could be indicative of a role for gene segment coevolution in adaptive niches such as landfowl and humans. However, it may instead be the case that coevolution between gene segments in the aquatic bird reservoir is species-specific. Improved surveillance of H9 viruses in migratory birds will be necessary to discern between these potential mechanisms.

In conclusion, our study reveals the importance of host niche in influenza virus evolution. It is clear that properties intrinsic to viruses do not always shape coevolution between gene segments in isolation, but that the host environment can alter the evolutionary trajectories taken. Further investigation of viral evolution in the context of virus-host coadaptation could reveal mechanistic insights into the factors governing viral evolution and emergence.

## MATERIALS AND METHODS

### Data collection and subsampling

Influenza A virus sequences were gathered and sampled as previously described (22). Briefly, FASTA files of genomic segments from avian H9 virus sequences were downloaded from the Influenza Research Database (IRD, http://www.fludb.org) (32) on 2 March 2021. FASTA files of protein coding sequences (CDS) for PB2, PB1, and PA from selected avian H9 virus sequences were downloaded from IRD on 30 June 2021. FASTA files of gene segments from human H9 virus sequences were downloaded from IRD on 24 September 2021. Human H3N2 virus sequences from the data set described in Jones et al*.* were used for comparative genomic analysis of avian H9 and human H3N2 viruses.

Sequences were read into R (version 4.1.0) using the DECIPHER package (version 2.20.0) (33). CDS were translated into amino acid sequences prior to alignment. Quality control was performed to ensure that sequence duplication, sequencing ambiguity, and incomplete genomes were excluded from the analysis. Concatenated alignments comprising all eight gene segments were used to construct species trees for avian H9 and human H3N2 virus sequences by clustering strains into taxonomic units by sequence identity (Fig. 1A). A sequence identity cutoff of 95% was selected for human H3N2 virus sequences on the basis that this yielded at least 10 clusters in the species tree (Fig. S1). Significant disparities were noted between human H3N2 and avian H9 virus cluster sizes at all cutoffs (e.g., 15 vs 200 respective clusters in human H3N2 and avian H9 viruses at a cutoff of 95%), so cutoffs of 90% and 95% identity were chosen for avian viruses to ensure that these studies were not biased by tree size. Sampling bias among avian H9 virus sequences was assessed by the year of isolation specified in the FASTA files of sequences after QC and again among sequences used to construct trees (Fig. S3). Gene and protein trees were built from randomly chosen cluster representatives. Gene trees can be found in Fig. S1 and S2.

### Phylogeography

Phylogeography was performed by analyzing gene trees of avian H9 viruses by continent of origin. All avian H9 strains that remained after QC (1,418 full-length sequences) were assigned to their continent of origin based on the isolation location specified in the FASTA file. Strains from ambiguous locations (e.g., “ALB”) were excluded. Sequences were available from all seven continents except Antarctica. These were mapped further to their country of origin and visualized on a world map using the following packages in R: rnaturalearth (version 0.1.0), rnaturalearthdata (version 0.1.0), rgeos (version 0.5-8), and sf (version 1.0-4). Only one sequence was available from Australia and was not analyzed further. Clustering was performed on avian H9 strains from the remaining five continents (Asia, Africa, Europe, North America, and South America) as described above, with a sequence identity cutoff of 96% selected for each. South American and African strains each clustered into fewer than 10 distinct clusters, so these continents were not analyzed further. Gene trees from the remaining three continents (Asia, Europe, and North America) were constructed from randomly chosen cluster representatives (Fig. S4 to S6).

### Host origin

Taxonomical orders represented in avian H9 virus sequences were determined based on hosts specified in FASTA files. Thirteen orders of the Aves class were identified: Galliformes, Anseriformes, Charadriiformes, Pelecaniformes, Gruiformes, Accipitriformes, Passeriformes, Strigiformes, Columbiformes, Falconiformes, Otidiformes, Struthioniformes, and Psittaciformes. Sequences with ambiguous or unspecified host species (e.g., “Avian”) were excluded. Sequences isolated from Galliformes spp. (including chickens, turkeys, quail, pheasant, guineafowl, and Chinese francolin) were designated as landfowl-derived (1,065 sequences). Sequences isolated from Anseriformes, Charadriiformes, Pelecaniformes, and Gruiformes spp. were collectively designated as aquatic bird-derived (300 sequences). Analysis of landfowl-derived and aquatic bird-derived H9 virus sequences was restricted to isolates from the Asian continent. Clustering was performed on landfowl-derived and aquatic bird-derived avian H9 sequences as described above, with a sequence identity cutoff of 94% selected for each. Similar numbers of clusters were found despite the considerable differences in overall strain numbers in each group (18 and 33 clusters for landfowl-derived and aquatic bird-derived species trees, respectively). Based on the low number of sequences available for human H9 viruses, all available sequences were used in the reconstruction of gene trees. Representative gene trees are shown in Fig. S7 to S9.

### Tree reconstruction

Maximum likelihood gene trees were built assuming a general time reversible model of nucleotide substitution using the ape (version 5.5) and phangorn (version 2.7.1) packages. Maximum likelihood protein trees were built assuming the HIV between-patient model (avian and human PB2 trees, human PA tree) or the FLU model (avian and human PB1 trees, avian PA tree) of amino acid substitution. Best-fit models were approximated by model testing using the AIC criteria. Where indicated by the best-fit model, rates were assumed to vary according to the proportion of invariant sites and/or the discrete Gamma distribution with four rate categories. All trees were assessed for bootstrap support using 1,000 replicates.

### Analysis of tree similarity

Tanglegrams, or back-to-back trees matching tips of two trees, were built from pairs of trees using the phytools package (version 0.7-80). The CID was calculated with the TreeDist package (version 2.1.1) (34). Statistical significance between tree distances was determined by the Mann-Whitney *U* test. Where multiple testing was performed, adjusted *P* values are reported after Benjamini-Hochberg *post hoc* correction.

## Data Availability

Revised code for analysis of coevolution in concatenated, full-length genomic influenza virus sequences is available on GitHub (https://github.com/Lakdawala-Lab/Host-Origin-and-Parallel-Evolution/). All human and avian FASTA files as well as individual phylogenetic trees are available on figshare (https://doi.org/10.6084/m9.figshare.c.7762478).

## SUPPLEMENTAL MATERIAL

Supplemental material jvi.01518-24-s0001.docx Table S1; Figures S1 to S9.

ASM does not own the copyrights to Supplemental Material that may be linked to, or accessed through, an article. The authors have granted ASM a non-exclusive, world-wide license to publish the Supplemental Material files. Please contact the corresponding author directly for reuse.
