# Supplementary material for: Host origin is a determinant of coevolution between gene segments of avian H9 influenza viruses
Source: J Virol. 2025 Jun 13;99(7):e01518-24. doi: 10.1128/jvi.01518-24 (PMC12282180; doi:10.1128/jvi.01518-24)

**Supplemental Information**

**Table S1. Strains analyzed in this study.**

| **Subtype** | **Host** | **Sequences After QC** | **Similarity Threshold** | **Sequences Analyzed** |
| --- | --- | --- | --- | --- |
| H3N2 | Human | 9,096 | 95% | 15 |
| H9 | Avian (all) | 1,258 | 90% | 56 |
|  |  |  | 95% | 200 |
|  | Avian (Asia) | 1,036 | 96% | 171 |
|  | Avian (Europe) | 19 | 96% | 12 |
|  | Avian (North America) | 115 | 96% | 61 |
|  | Landfowl (Asia) | 1,018 | 94% | 56 |
|  | Aquatic birds (Asia) | 139 | 94% | 31 |
|  | Human (Asia) | 10 | N/A | 10 |


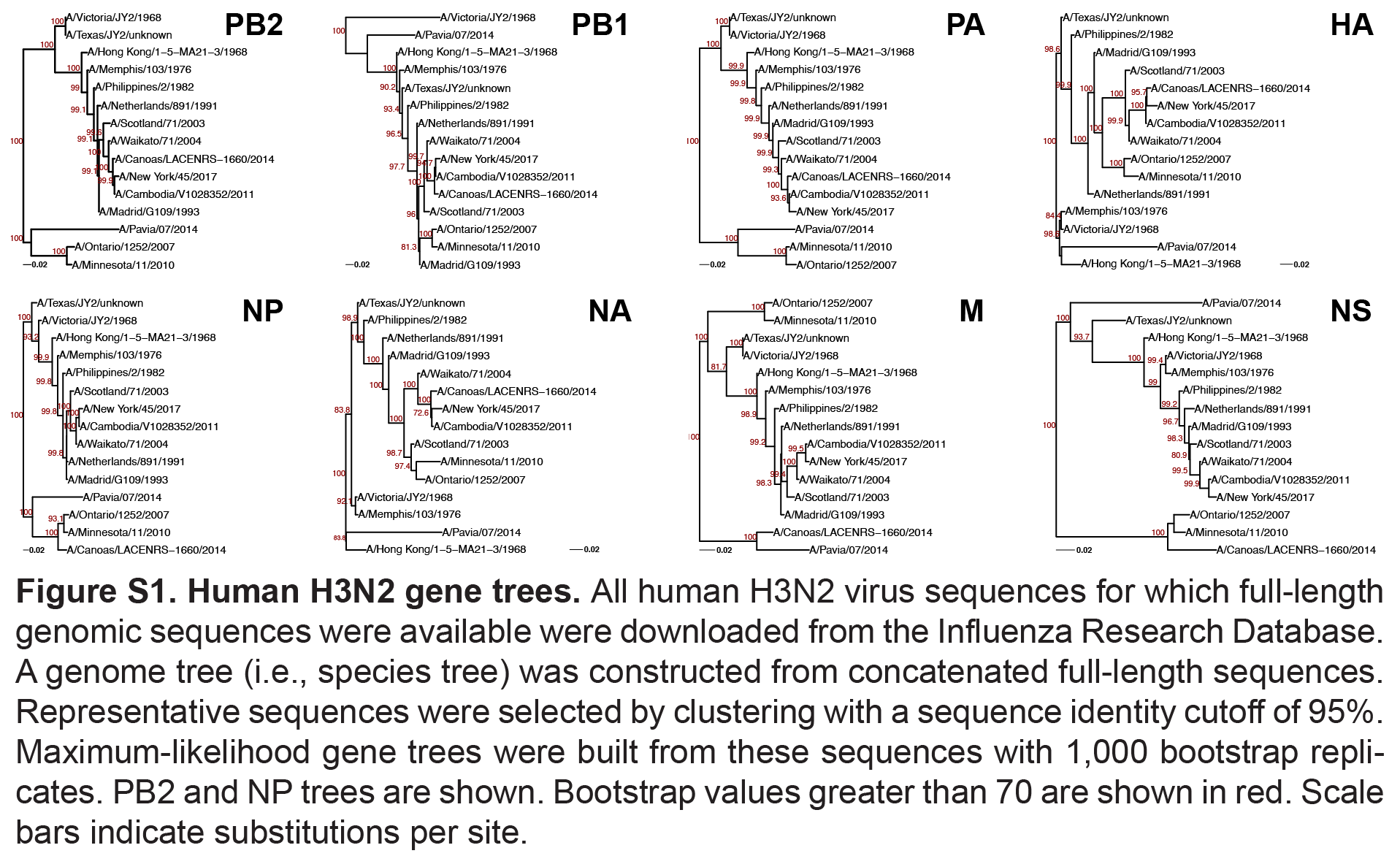


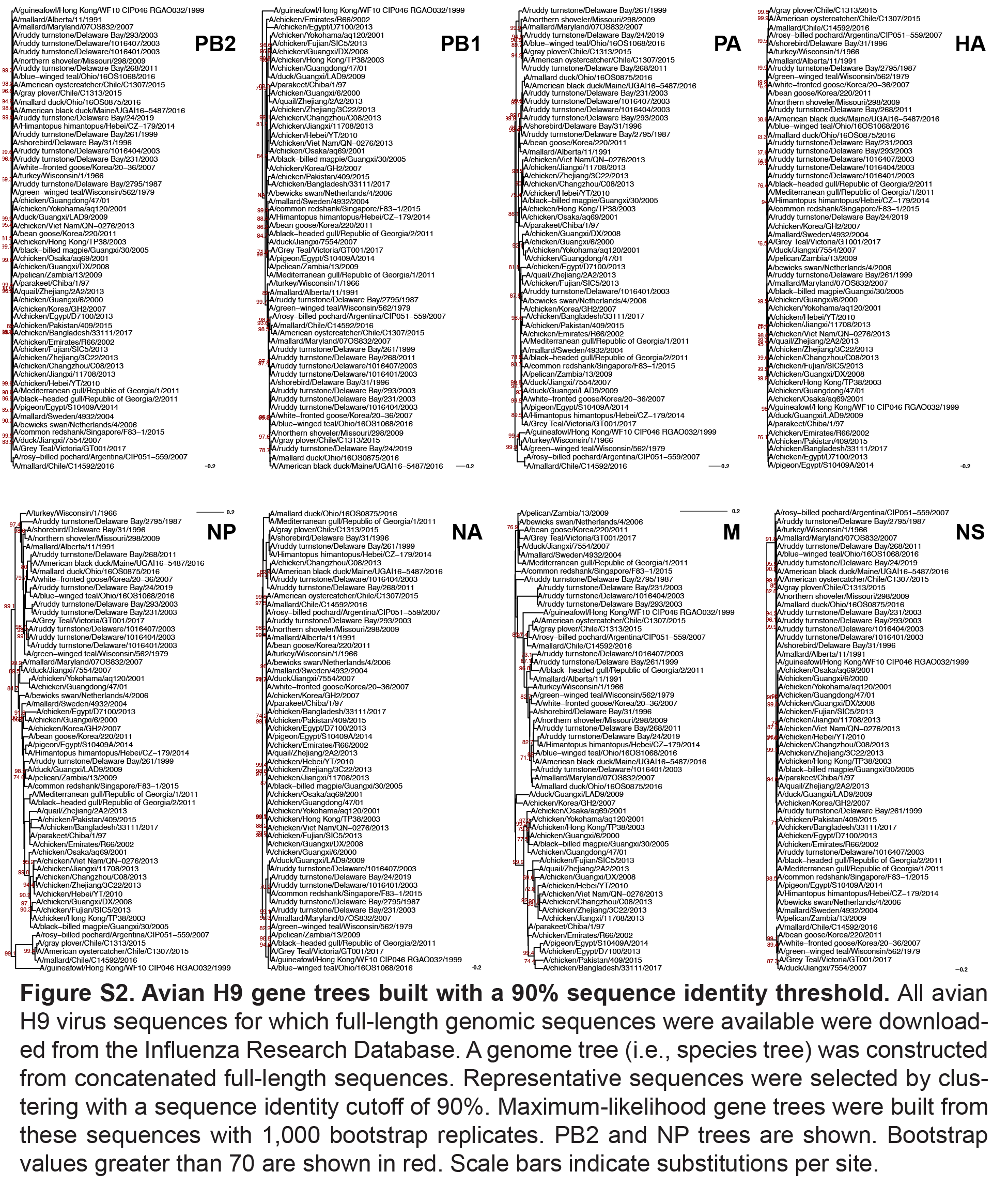


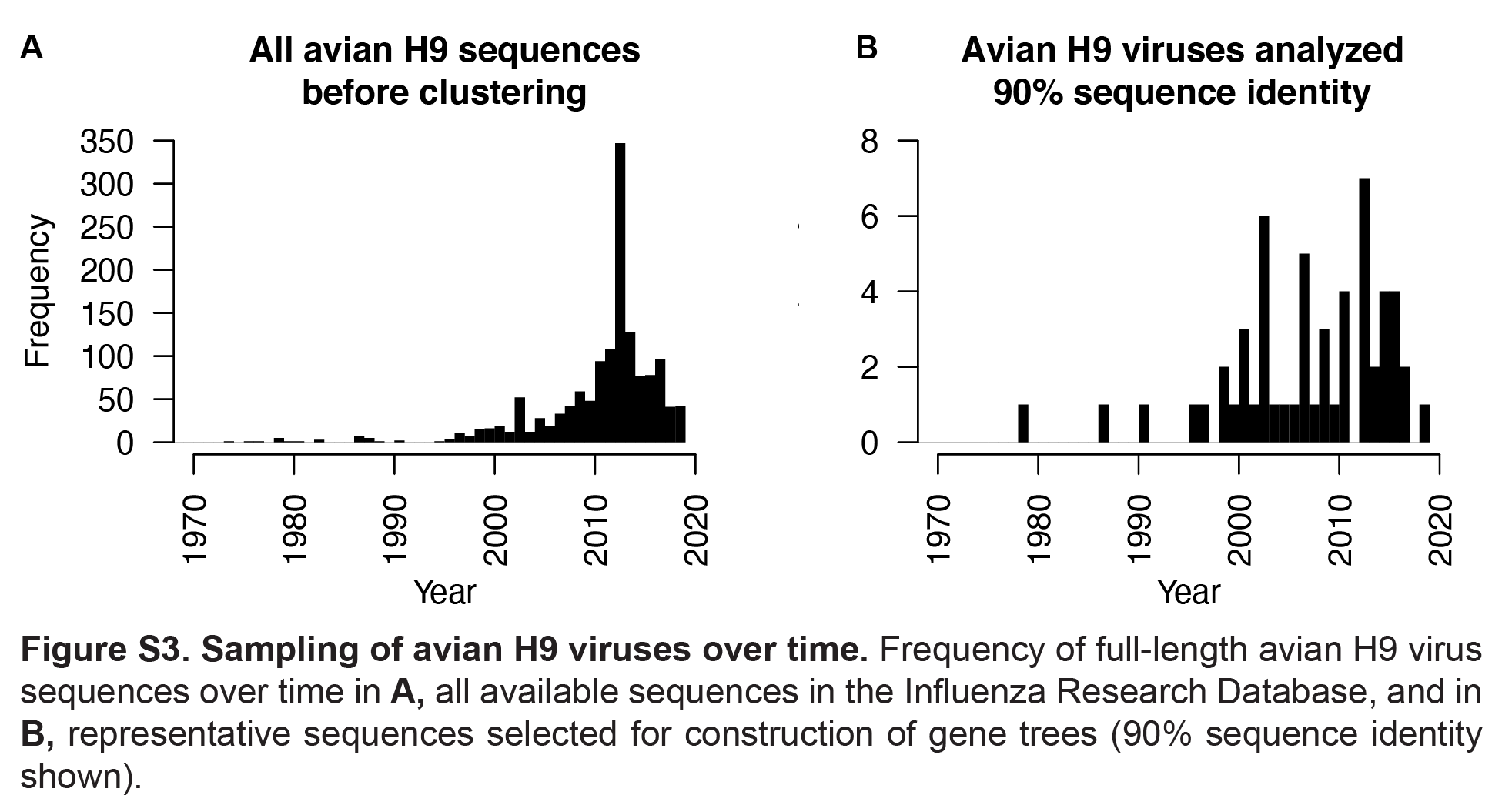


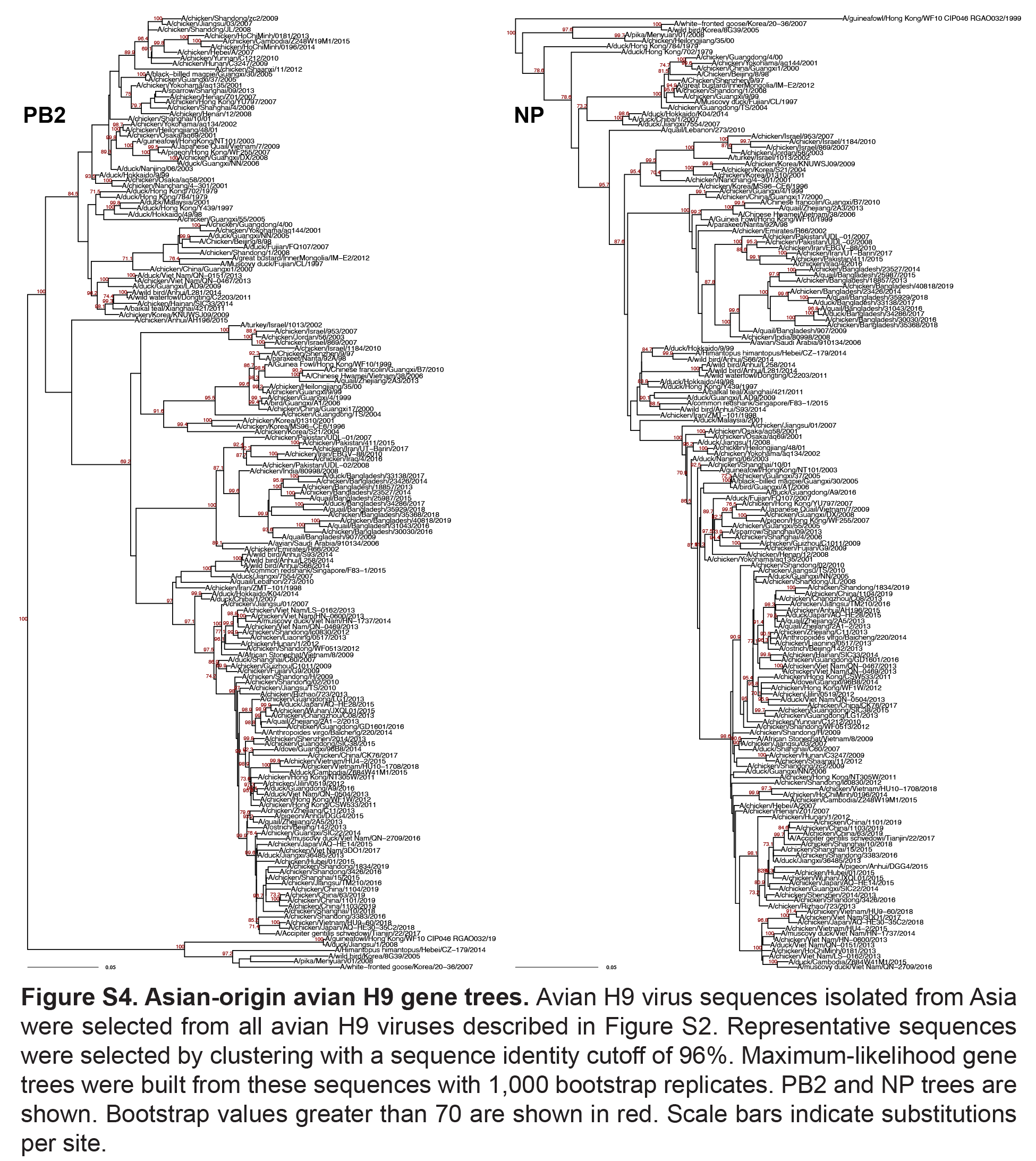


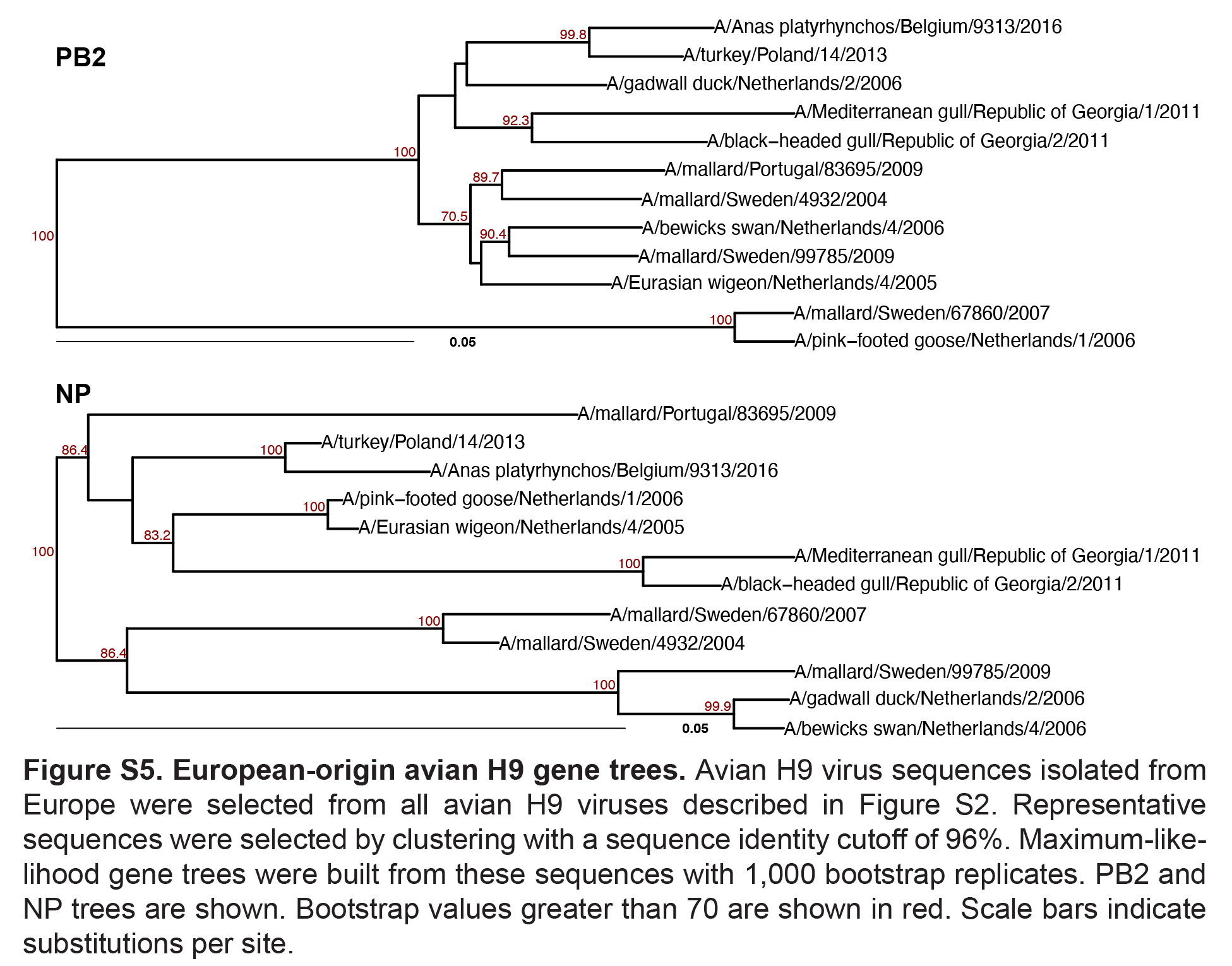


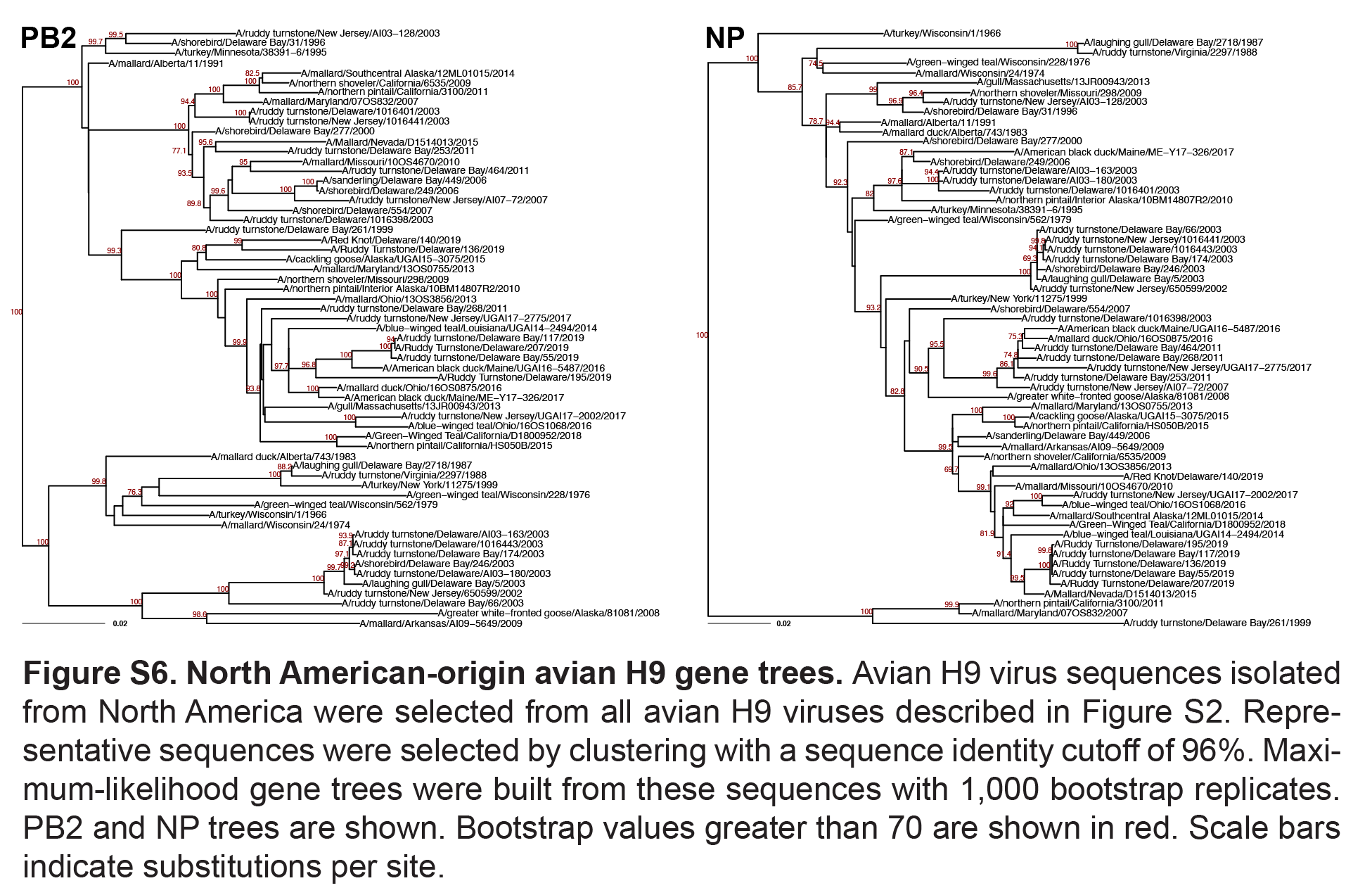


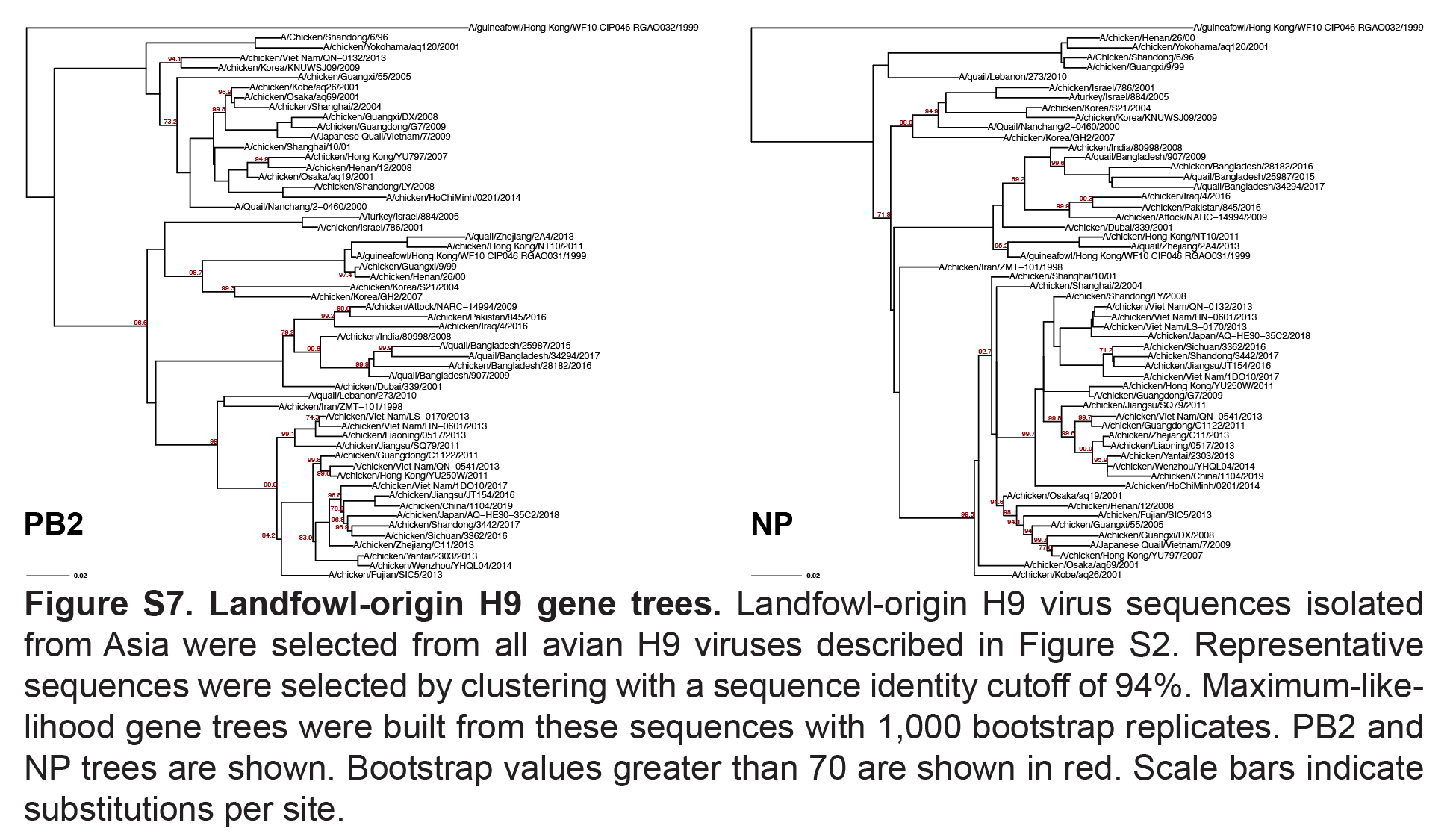


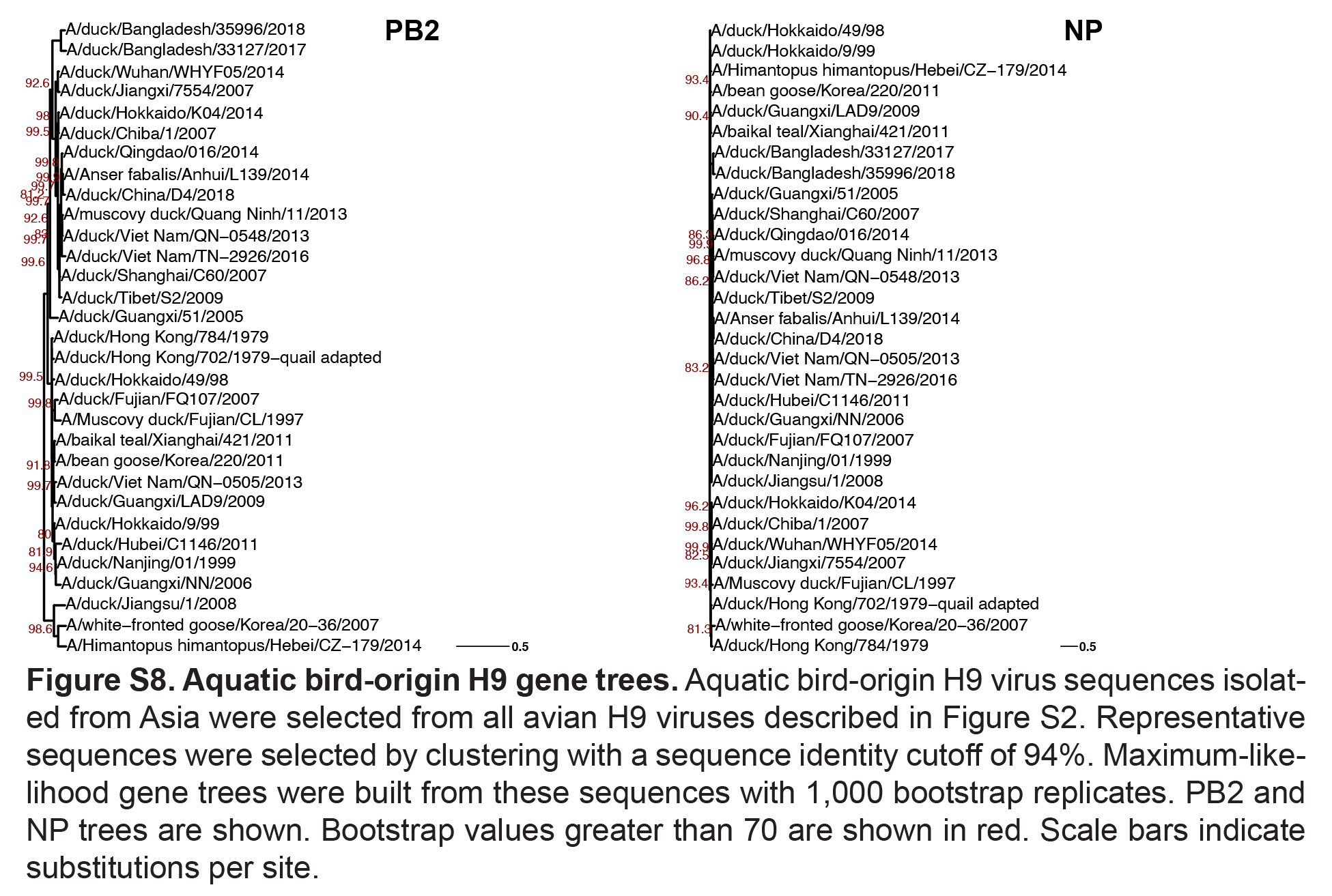


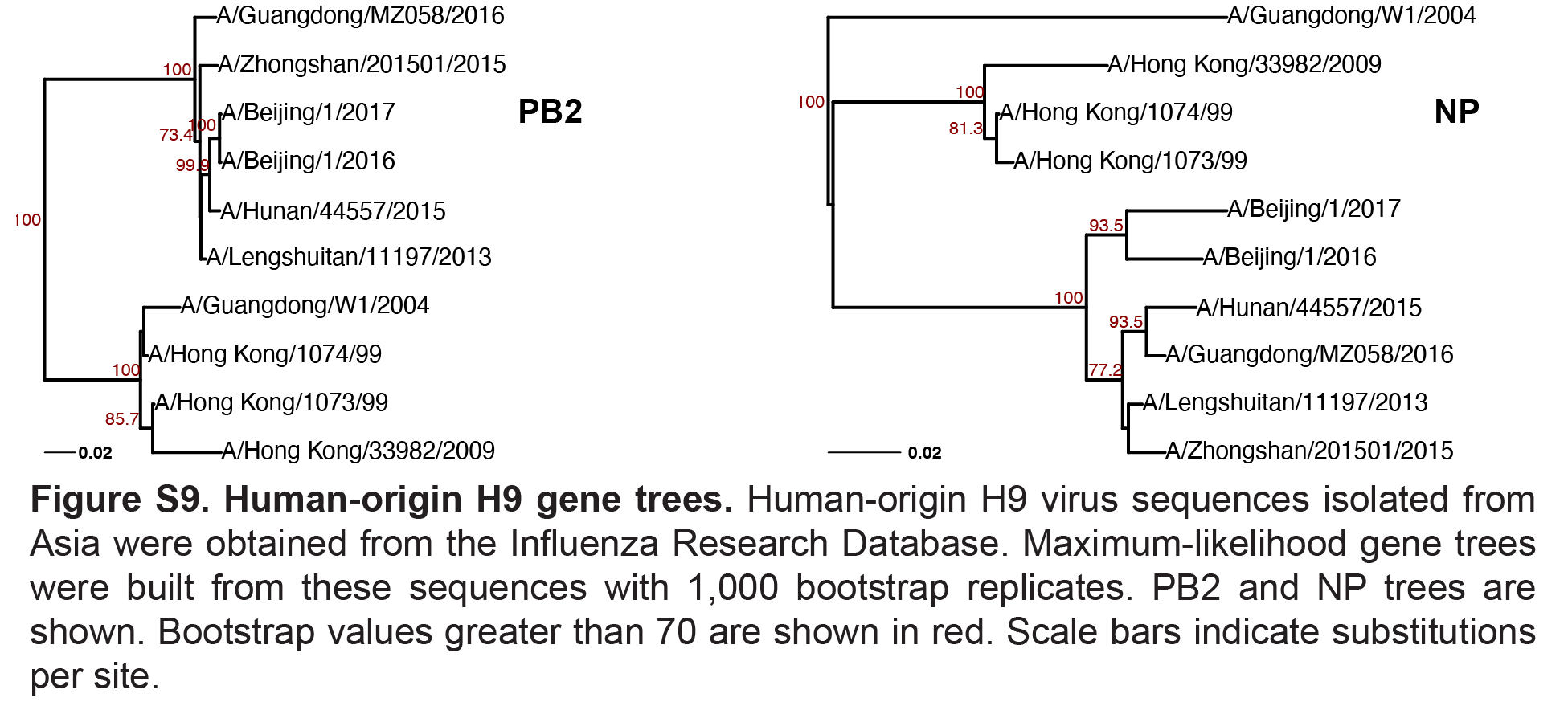

Supplement: Supplemental material — Table S1; Figures S1 to S9. [file jvi.01518-24-s0001.docx]
